# Assessing genotype–environment interactions in Atlantic salmon reared in freshwater loch and recirculating systems

**DOI:** 10.1111/eva.13751

**Published:** 2024-08-09

**Authors:** Mette J. Tollervey, Michaël Bekaert, Agustín Barría González, Saif Agha, Ross D. Houston, Andrea Doeschl‐Wilson, Ashie Norris, Herve Migaud, Alejandro P. Gutierrez

**Affiliations:** ^1^ Institute of Aquaculture University of Stirling Stirling UK; ^2^ Cooke Aquaculture Scotland, Avondale House, Strathclyde Business Park Bellshill UK; ^3^ Roslin Institute The University of Edinburgh Edinburgh UK; ^4^ Animal Production Department, Faculty of Agriculture Ain Shams University Shubra Alkhaima, Cairo Egypt; ^5^ Benchmark Genetics, Edinburgh Technopole Edinburgh UK; ^6^ Mowi Genetics AS Bergen Norway; ^7^ Mowi Scotland, Glen Nevis Business Park Fort William UK

**Keywords:** aquaculture, breeding systems, GxE, phenotypic plasticity, *Salmo salar*

## Abstract

The interest in recirculating aquaculture systems (RAS) is growing due to their benefits such as increased productivity, better control over animal care, reduced environmental effects, and less water consumption. However, in some regions of the world, traditional aquaculture methods remain prevalent, and selective breeding has often been designed for performance within these systems. Therefore, it is important to evaluate how current fish populations fare in RAS to guide future breeding choices. In a commercial setting, we explore the genetic structure of growth characteristics, measure genotype–environment interactions (GxE) in salmon smolts, and examine genetic markers related to growth in freshwater lochs and RAS. Young salmon were raised together until they reached the parr stage, after which they were divided equally between freshwater net‐pens and RAS. After an 8‐week period, we sampled fish from each environment and genotyped them. Our findings revealed that fish reared in RAS were generally smaller in weight and length but exhibited a higher condition factor and uniformity. We found a notably smaller component of unexplained variance in the RAS, leading to higher heritability estimates. We observed a low GxE effect for length and condition factor, but significant re‐ranking for whole‐body weight, as well as noticeable differences in trait associations across environments. Specifically, a segment of chromosome 22 was found to be linked with the condition factor in the RAS population only. Results suggests that if the use of RAS continues to expand, the efficiency of existing commercial populations may not reach its full potential unless breeding programs specific to RAS are implemented.

## 1 INTRODUCTION

The Atlantic salmon, *Salmo salar* L. 1758, is an important species in the aquaculture, with Norway, Chile and the United Kingdom the largest producers globally (FAO, 2022). Recent decades have witnessed an increasing shift towards land‐based systems, in particular, fully closed recirculating aquacultural systems (RAS), motivated by advantages this system offers (Bergheim et al., 2009). These include its scalability, site versatility, product consistency, biosecurity, and environmental stability (Ebeling & Timmons, 2012; Kolarevic et al., 2014; Mota et al., 2019). The system's capacity to recycle water also minimise land and water usage and lessens issues associated with escaped fish, disease, and waste (Ebeling & Timmons, 2012; Thorarensen & Farrell, 2011). However, this process requires mechanical, chemical, and biological filtration to prevent water chemistry changes during fish rearing (Ebeling & Timmons, 2012). Consequently, due to the substantial cost of establishing and operating RAS, fish are often reared at higher densities to maintain profitability; provided the system can adequately process the increased concentrations of fish derived waste (Thorarensen & Farrell, 2011).

In Scotland, however, which is the main salmon production within the United Kingdom, freshwater lochs with floating net‐pens are primarily used until the salmon reach the smolt stage (Bergheim et al., 2009; Houston et al., 2020). In contrast to RAS, these loch‐based systems are cost‐effective, and simpler to operate, but allow limited control over conditions and are susceptible to environmental factors (Ellis et al., 2016). But in line with other countries, there is a growing interest in adopting RAS in Scotland (Bostock et al., 2018; Clarke & Bostock, 2017). However, current commercially used salmon lines have been selected based on their performance in loch net‐pens during their freshwater phase. Therefore, if RAS is to be more broadly adopted, assessment of the relative performance of selected stocks in both environments is necessary.

A potential strategy to compare the performance of different fish families across various environments involves exploring the genotype–environment interactions (GxE), which refers to phenotypic changes in different environments for a given genotype (Falconer, 1952; Falconer & Mackay, 1996). GxE is typically identified by examining variations in genetic parameters, including heritability, genetic variance, and genetic correlations amongst traits within each environment (Sae‐Lim et al., 2014). A significant concern for aquaculture is re‐ranking, where the top‐performing genotype in one environment does not perform equally well in another (Mulder et al., 2006; Sae‐Lim et al., 2016). If present, GxE will limit the effectiveness of breeding programmes unless addressed (Mulder et al., 2006; Mulder & Bijma, 2005).

Re‐ranking of genotypes is typically viewed in terms of the genetic correlation between the same trait measured in different environments (Mulder et al., 2006; Sae‐Lim et al., 2016). While values that deviate from 1 indicate re‐ranking, where these fall below 0.8 GxE is considered to be of biological significance to a program of selection (Robertson, 1959). In more recent years, studies have proposed ‘break‐even correlations’ which help assess when use of sib‐testing, index selection or environment specific breeding would be of more advantage compared to a single breeding program (Sae‐Lim et al., 2016). For livestock, these estimates have ranged from 0.61 to 0.7 (James, 1961; Mulder et al., 2006). Recently, a new benchmark of 0.7 for aquacultural species has been suggested (Sae‐Lim et al., 2013). Estimates from other species comparing performance in RAS to other systems have generally revealed significant estimates of GxE, with genetic correlations ranging from 0.65 to 0.27 (Fernandes et al., 2019; Li et al., 2019; Mas‐Muñoz et al., 2013; Sae‐Lim et al., 2014; Van Sang et al., 2020), although some studies have reported lower GxE (Dupont‐Nivet et al., 2008, 2010; Turra et al., 2016). However, similar studies comparing freshwater environments used in Atlantic salmon aquaculture are yet to be conducted.

This study aims to bridge this gap by investigating the genotype–environment interactions in RAS and loch environment on Atlantic salmon growth during their freshwater development within a commercial context. Our objectives include: estimating genetic parameters and heritability for Atlantic salmon within each rearing environment; determining GxE by calculating the genetic correlation between growth traits when measured in each environment; and comparing genetic markers associated with growth traits between environments. The insights from this research will be instrumental in guiding future breeding strategies and husbandry decisions in the context of the growing utilisation of RAS.

## 2 MATERIALS AND METHODS

### 2.1 Source population

The study population came from the nucleus family breeding programme of Mowi Ireland 2022 Generation where each year, nucleus and dissemination families are produced from approximately 163 dams and 90 sires. In brief, for the study population, broodstock were spawned over two subsequent days. Families were produced mostly using a hierarchical mating structure, where one male is used to inseminate the eggs of two females. Breeding goals for this nucleus population focused on: resistance to Cardiomyopathy syndrome (CMS), growth, and lower sexual maturity. Eggs were evacuated from sacrificed dams using air before separate incubation until hatching (at 400 degree‐days). Eggs were then combined into groups, maintain the same number of eggs per dam.

### 2.2 Experimental design

In January 2021, 250,000 eyed eggs from 150 families in the nucleus population were transferred to recirculating aquaculture facilities (RAS) in the northwest of Scotland. From first feeding, fish were kept in a single fry unit for 5 weeks before being split between two fry tanks (116,500). After 3 weeks, the fish from both fry tanks were reallocated across four fry tanks, before the number of fish in each tank was approximately evened out to 52,000. After a further 6 weeks the population was again transferred into two smolt tanks (96,500 in each) at which point a bottom 5% cull was performed (11,197 fish removed). Fish from the remaining two smolt tanks underwent vaccination over two consecutive days in September 2021. The population was then placed back into 10 fry tanks (19,000 each) with a further bottom 4.7% (8961 fish removed) cull performed.

Before approximately half the population was transferred to freshwater (FW) loch site, fish were sexed and split by sex via use of ultrasound. In the middle of sexing and as soon as there were enough males and females for transportation to the FW loch site (22nd of September 2021), three tanks of males and three tanks of females were moved. At this site, all females and all males were pooled (such that there was a single tank per phenotypic sex). This corresponded to 91,708 fish (32,584 females and 59,124 males). The remaining population in the RAS facilities included 91,713 fish (45,874 females and 45,839 males) split between six fry tanks, three male and three female, with 15,000 smolts in each. Fish remained in these tanks/pens until transfer to SW site for grow out. In RAS facilities, tanks were 50 m^3^, in the loch environment pens had a capacity of 2048 m^3^. This corresponded to a density of 300 fish/m^3^ and 22 fish/m^3^ in RAS and loch environment respectively. Fish remained in these freshwater environments until November 2021 when they were sampled.

In RAS, parr were initially held under 12 h light/darkness (LD) cycles. In October 2021, the population remaining in RAS were placed under continuous light (24 h light, LL). In land‐based systems, parr are often exposed to continuous light (LL) as it enhances growth performance. However, for parr to undergo smoltification they require both exposure to winter short day (LD) and then return to and increase or long day (LL) photoperiod (Björnsson et al., 2000).

In both environments, measurements of water temperature, pH, and oxygen saturation were taken daily. Additional water quality parameters were recorded in the RAS environment. Specifically, carbon dioxide, total ammonia and nitrate (TAN), ammonia (NH_3_), nitrite $\left({\mathrm{NO}}_{2}^{-}\right)$, nitrate $\left({\mathrm{NO}}_{2}^{-}\right)$ concentration, total alkalinity (CaCO_3_), hardness and, turbidity.

### 2.3 Phenotypic traits

A total of 1000 fish were sampled per environment at the end of the freshwater rearing (after a total of 56 days), at approximately 10 months of age. All fish were sacrificed following administration of lethal dose of anaesthesia as per the schedule 1 protocol, UK Animals (Scientific Procedures) Act 1986 Amended Regulations (SI 2012/3039) Animal Welfare. Measurements of whole‐body weight (WBW) and length (tip of head, snout, to the deepest point of the fork in the caudal fin) were recorded, from which condition factor (K = W/L^3^) was calculated. Additionally, fin clips were taken from the adipose fin of all sampled fish for genotyping and pedigree reconstruction.

### 2.4 Genotyping and pedigree reconstruction

The parental broodstock population were previously genotyped to 55 K SNP (non‐public Axiom array, NOFSAL03). Sampled fin clip of the study (offspring) population were genotyped to 66 K SNP by IdentiGEN Ltd (non‐public Axiom array, SALMOWI). SNPs were called based on major allele frequency with Applied Biosystems – Analysis Power Tools (APT) v2.11.6. Pedigree reconstruction and family assignment was performed by MOWI using the sequenced genotypes and their own in‐house software, which employs an opposite homozygosity (OH) method. Specifically, between all the sires and dams mated to produce the study population, the OH was counted when the broodstock genotypes were compared to that of each of the sampled offspring. Sire and dams with the lowest OH were assigned temporarily as a parent. Sire and dam combinations based on OH were then compared to the list of known matings recorded by MOWI. When sires and dams did not appear in the know mate pairings, the parentage was rejected, and a likelihood approach was use for those offspring.

For further analysis, broodstock and offspring genotypes were filtered. Using PLINK v1.9 (Purcell et al., 2007), duplicated and unaligned SNPs were removed in both offspring and broodstock genotypes. Remaining SNPs were filtered, removing those that did not meet the following criteria: individuals whose more than 10% of genotypes were missing, SNPs that were missing more than 10% of individuals' genotypes, SNPs that failed to meet Hardy–Weinberg equilibrium (*p*‐value > 10^−6^), and SNPs with minor allele frequency lower than 0.005. Common alleles were extracted and used for further analysis.

### 2.5 Statistical analysis

Normality was assessed from histograms of raw data and q‐q plots post statistical testing. Mean and standard error were calculated for each trait by genotypic sex, environment, and genotypic sex within environment. Effects of genotypic sex and environment (RAS and Loch), with nested effect of tank or pen, on trait averages and variance were investigated through two‐way ANOVA with Post Hoc Tukey test (*p*‐value > 0.05). All statistics were evaluated with R v4.2.2 (R Core Team, 2022).

### 2.6 Univariate analysis

All models and genomic analysis were performed in BLUPF90 software release 2023‐04‐15 (Misztal et al., 2015). Within each environment, a univariate animal model (Equation 1) was fit via implementation of a restricted maximum likelihood (REML) approach:

(1) Y=Xb+Zu+e

where, *Y* is a vector of phenotypic records of the population. *X* is a design matrix linking individuals to the vector of fixed effects. Vector *b* represent the genotypic sex and tank/pen. *Z* is a design matrix linking individuals to a vector of additive genetic effect *u*. This was firstly estimated from the pedigree (*A* matrix), where *u* has a normal distribution $∼N\left(0,V_{g}\times A\right)$, and *A* is the numerator relationship matrix and *V*_*g*_ is additive genetic variance. Secondly, parameters were estimated using the genomic data, where $u\;∼\;N\left(0,V_{g}\times \mathrm{GRM}\right)$, and *GRM* is the genomic relationship matrix. Single step genomic evaluation was also performed where $u\;∼\;N\left(0,V_{g}\times H\right)$. In this, the *H* matrix combines information from both pedigree (*A* matrix) and SNP data (*GRM* matrix), as defined in Legarra et al. (2009). Lastly, *e* is a vector of residual effects with $e\;∼\;N\left(0,V_{r}\times I\right)$, where *I* is the identity matrix and *V*_*r*_ is residual variance. For analysis, full pedigree and SNP data was provided but using only the phenotypic data of this specific rearing environment.

For each trait, narrow sense heritability(*h* ^2^) was calculated as *V*_*g*_ */V*_*p*_, where *V*_*p*_ is phenotypic variance (*V*_*g*_+*V*_*r*_). Heterogeneity of trait variances between environments was compared by calculating the coefficient of phenotypic ($\mathrm{CV}=\left({\mathrm{SD}}_{p}/\text{mean}\right)\times 100$) and genetic $\left(\mathrm{CGV}=\left({\mathrm{SD}}_{g}/\text{mean}\right)\times 100\right)$ variances (Sae‐Lim et al., 2014).

### 2.7 Multivariate analysis

Within each environment, a multivariate model was performed (Equation 2), and pairwise combinations of the three growth traits, *Y*_1_ and *Y*_2_, were simultaneous fitted:

(2) $$\left(\begin{array}{c} Y_{1}\\ Y_{2} \end{array}\right)=\left(\begin{array}{c} X_{1}\;0\\ 0\;X_{2} \end{array}\right)\left(\begin{array}{c} b_{1}\\ b_{2} \end{array}\right)\;+\;\left(\begin{array}{c} Z_{1}\;0\\ 0\;Z_{2} \end{array}\right)\left(\begin{array}{c} u_{1}\\ u_{2} \end{array}\right)\;+\;\left(\begin{array}{c} e_{1}\\ e_{2} \end{array}\right)$$

In which, the same effects were fitted as univariate and bivariate analysis. From estimated genetic and residual (co) variances, the genetic (*r*_*g*_) and residual (*r*_*r*_) correlations between traits were calculated.

### 2.8 Genotype–environment interaction (GxE)

Estimates of GxE were obtained from a similar multi‐trait model as described above. However, here, the same trait measured in each of the environments was treated as two independent traits. The strength of GxE was then quantified by the genetic correlation (*r*_*g*_) between the two traits (Mulder & Bijma, 2005; Sae‐Lim et al., 2016). Fixed effects of genotypic sex within RAS environment, genotypic sex within loch environment, and tank/pen were fit. Residual covariance was set to zero, as each fish could only inhabit in a single environment:

(3) $$\left(\begin{array}{c} e_{1}\\ e_{2} \end{array}\right)\;∼\;\mathrm{MVN}\left(I\times \left(\begin{array}{cc} \sigma_{r,1}^{2} & 0\\ 0 & \sigma_{r,2}^{2} \end{array}\right)\right)$$

### 2.9 GWAS

Genome‐wide association (GWA) was performed in GCTA v1.940 (Yang et al., 2011). A mixed linear model approach, following a leave‐one‐chromosome‐out principle. This was fit as Equation 1, with the same fixed and random effects and using the same set of filtered SNPs and G matrix as both univariant and bivariant analysis (Equations 1 and 2). A 5% significant threshold was calculated using Bonferroni correction at both the genome and chromosome level. These were set based on the total number of SNPs, $-\log_{10}\left(0.05/\mathrm{SNP}\;\text{number}\right)$, as well as the average number of SNPs per chromosome, $-\log_{10}\left(0.05/\mathrm{SNP}\;\text{number}/\mathrm{Nb}.\;\text{Chromosome}\right)$, respectively.

## 3 RESULTS

### 3.1 Environmental description

**FIGURE 1:**
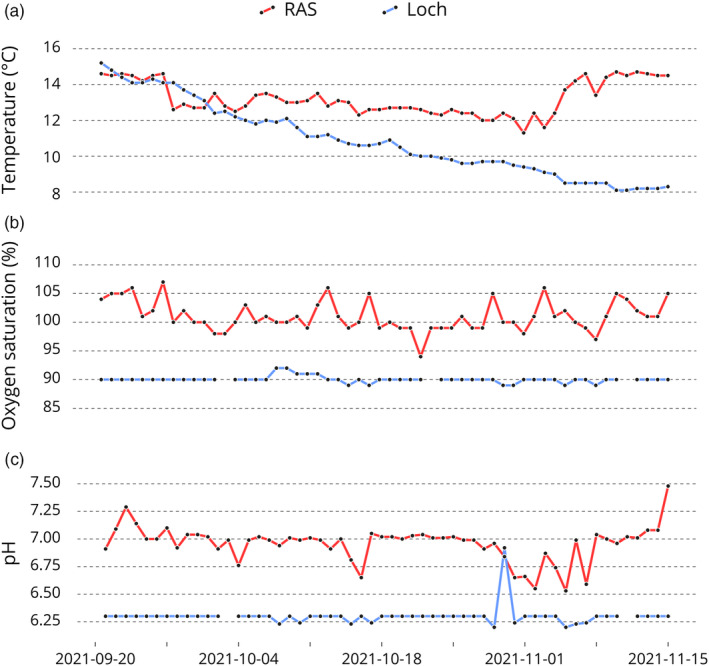
Temperature (a), oxygen saturation (b) and pH measurement (c) for the 56 days of the study in the RAS (red) and loch (blue) freshwater environments.

The main differences in environmental conditions can be seen in Figure 1. The Recirculating Aquaculture System exhibited a higher and more stable mean water temperature of 13.21°C (standard deviation, 0.94°C) in comparison to the loch environment, which stood at 10.86°C (Standard deviation, SD 2.04°C) and displayed a decreasing trend over time. Oxygen saturation in the RAS was notably higher (101.09%, SD 2.64%) compared to the loch environment (90.02%, SD 0.57%), though the loch environment showed reduced fluctuation. Finally, pH levels were observed to be more acidic in the loch environment (6.30, SD 0.09) versus the RAS environment (6.96, SD 0.16), which also had less variability. More water quality parameters from the RAS are available in Table S1.

### 3.2 Population structure

In both environments, the mortality rate was low. The number of mortalities in the RAS environment was 2657 corresponding to 2.90%. In the loch environment this value was only slightly greater at 3836 mortalities, corresponding to a loss of 4.18%. A total of 2000 fish were sampled. After QC, genotypic data was obtained for 1942 offspring. A total of 55,357 SNPs were identified in the parent genotypes and 65,774 in the offspring, with 53,489 and 59,578 SNPs remaining post‐filtering, respectively (Table S2). There were 45,751 SNPs that were common to both parents and offspring populations (Table S3 and Data S1).

Pedigree reconstruction identified 72 sires and 139 dams in the parental pool. There were 65 offspring that could not be matched to a sire and out of these, 41 also could not be matched to a dam. In total, 141 full‐sib (including two families with unassigned sire), 72 sire (half‐sib) and 139 dam (half‐sib) families were identified, with 134, 71 and 134 families, respectively, being common across both environments. Though the average full‐sib family size was 6.8 and 6.9 in the RAS and loch environments, respectively, this was variable and ranged from 1 to 28. Furthermore, for each full‐sib family its size was significantly different between environments (Chi‐squared statistic = 308.59, *df* = 132, *p*‐value = 3 × 56^−16^), with the difference between environments ranging from −18 to 14.

### 3.3 Phenotypic parameters

Smolts from the loch environment were significantly heavier (degrees of freedom = 1, *F*‐value = 1047.08, *p*‐value < 2 × 10^−16^) and longer (*df* = 1, *F*‐value = 1219.39, *p*‐value < 2 × 10^−16^) than their RAS counterparts (Figure S1). However, RAS smolts displayed a greater condition factor than those from the loch environment (*df* = 1, *F*‐value = 27.33, *p*‐value = 1.90 × 10^−7^). Though at a lower magnitude, sex had an additional impact on weight (*df* = 1, *F*‐value = 40.32, *p*‐value = 2.67 × 10^−10^) and length (*df* = 1, *F*‐value = 38.04, *p*‐value = 8.41 × 10^−16^), with male smolts being, on average, 6.57 g (SD 4.52–8.61) lighter and 0.404 cm (SD 0.27–0.54) shorter than female smolts. There was no discernible sex effect on the condition factor. Furthermore, no evidence of a sex‐by‐environment interaction was noted across all traits (WBW: *df* = 1, *F*‐value = 0.62, *p*‐value = 0.43; length: *df* = 1, *F*‐value = 0.30, *p*‐value = 0.71; condition factor: *df* = 1, *F*‐value = 1.02, *p*‐value = 0.31). However, the tank/pen did have a significant impact but only within the RAS environment (WBW: *df* = 2, *F*‐value = 19.17, *p*‐value = 5.73 × 10^−9^; length: *df* = 2, *F*‐value = 46.93, *p*‐value < 2 × 10^−16^; condition factor: *df* = 2, *F*‐value = 49.36, *p*‐value < 2 × 10^−16^). While larger, loch‐reared fish exhibited higher standard deviation and coefficient of variance than RAS fish across all traits (Table 1 and Data S2).

**TABLE 1:** Descriptive statistics for both the overall and environment specific populations, where WBW is whole body weight, K is condition factor, *CV* the coefficient of variation, *V*_*r*_ the component of residual variance, *V*_*g*_ the component of genetic variance, *CGV* the coefficient of genetic variance and *h* ^2^ estimated heritability.

	WBW (g)		Length (cm)		K	
	RAS	Loch	RAS	Loch	RAS	Loch
Mean (SE)	88.03 (0.57)	121.87 (0.87)	19.05 (0.04)	21.35 (0.05)	1.26 (0.30 × 10^−2^)	1.24 (0.40 × 10^−2^)
*CV* %	20.30	22.24	6.55	7.83	6.67	8.80
*V*_*r*_ (SE)	175.08 (13.26)	591.94 (35.11)	0.83 (0.06)	2.33 (0.14)	0.38 × 10^−2^ (0.27 × 10^−3^)	1.00 × 10^−2^ (0.59 × 10^−3^)
*V*_*g*_ (SE)	127.10 (21.17)	129.58 (33.26)	0.55 (0.10)	0.43 (0.12)	0.22 × 10^−2^ (0.39 × 10^−3^)	0.15 × 10^−2^ (0.50 × 10^−3^)
*CGV* %	12.81	9.34	3.91	3.06	3.76	3.15
*h* ^2^ (SE)	0.42 (0.06)	0.18 (0.04)	0.40 (0.06)	0.15 (0.04)	0.37 (0.05)	0.13 (0.04)

### 3.4 Genetic variance and trait heritability

The *A* matrix is the numerator relationship matrix based on pedigree information only, while the *GRM* is the genomic relationship matrix based on genotypic SNP data, and the *H* matrix combines information from both. The estimates of genetic parameters from *H* or *GRM* matrix models were largely comparable, whereas the *A* matrix model showed lower and more variable variance, heritability, and genetic correlation estimates. For the *H* matrix (Table 1), 0.67% of genotypes were absent. The correlation between the diagonal elements of *GRM* and *A* matrices stood at 0.57, while the correlation of the off‐diagonal elements was marginally below the 0.5 threshold, at 0.43. Details for the genetic parameter estimates derived from *A* and *GRM* matrices in the Tables S4 and S5.

For all traits, the loch environment showed a significantly higher residual variance than the RAS environment. In the case of WBW, the genetic variance estimates did not differ across environments. Conversely, for length and condition factor traits, the RAS environment displayed greater genetic variance (Table 1). This translated to markedly higher heritability estimates in the RAS compared to the loch environment. More precisely, heritability estimates were moderate‐to‐high in the RAS (0.37–0.42) and low‐to‐moderate in the loch (0.13–0.18) reared populations. Likewise, the coefficient of genetic variance was larger for the RAS population across all traits (Table 1).

### 3.5 Within environment genetic correlations

The genetic, residual, and phenotypic correlations amongst growth traits in each environment are presented in Table 2. In both environments, significantly positive genetic, residual, and phenotypic correlations were observed between WBW and length, approximating one. Similarly, significantly positive correlations were seen between WBW and condition factor, but at a lower magnitude. Of note, the correlations recorded in RAS tended to be greater than that reported in the loch environment, apart from the phenotypic correlation between WBW and condition factor.

**TABLE 2:** Genetic (*r*_*g*_), residual (*r*_*r*_) and phenotypic (*r*_*p*_) correlations between growth traits within RAS and loch environments.

	Environment	*r*_*g*_ (SE)	*r*_*r*_ (SE)	*r*_*p*_ (SE)
WBW‐Length	RAS	**0.95 (0.12 × 10** ^**−1**^ **)**	**0.94 (0.58 × 10** ^**−2**^ **)**	**0.95 (0.41 × 10** ^**−2**^ **)**
	Loch	**0.94 (0.36 × 10** ^**−1**^ **)**	**0.92 (0.66 × 10** ^**−1**^ **)**	**0.92 (0.51 × 10** ^**−2**^ **)**
WBW‐K	RAS	**0.350 (0.11)**	**0.28 (0.48 × 10** ^**−1**^ **)**	**0.31 (0.35** ^**−1**^ **)**
	Loch	**0.28 (0.23)**	0.14 × 10^−1^ (0.42 × 10^−1^)	**0.53 × 10** ^**−1**^ **(0.33 × 10** ^**−1**^ **)**
Length‐K	RAS	0.37 × 10^−1^ (0.13)	−0.34 × 10^−1^ (0.52 × 10^−1^)	−0.69 × 10^−2^ (0.38 × 10^−1^)
	Loch	−0.36 × 10^−1^ (0.26)	**−0.37 (0.36 × 10** ^**−1**^ **)**	**−0.33 × 10** ^**−1**^ **(0.29 × 10** ^**−1**^ **)**

*Note*: Values different from zero are indicated in bold.

On the other hand, the correlations estimated between length and condition factor differed between environments. Specifically, the loch environment demonstrated significantly negative residual and phenotypic correlations between length and condition factor. In contrast, positive correlations were observed between these two variables in the RAS environment, though the error estimates overlapped zero.

### 3.6 GxE

The genetic correlations (*r*_*g*_) hint towards a range of moderate to weak GxE effects, with the strongest effect seen in WBW (0.62, Standard error, SE 0.14), followed by length (0.78, SE 0.15) and condition factor (0.85, SE 0.17).

### 3.7 GWAS

**FIGURE 2:**
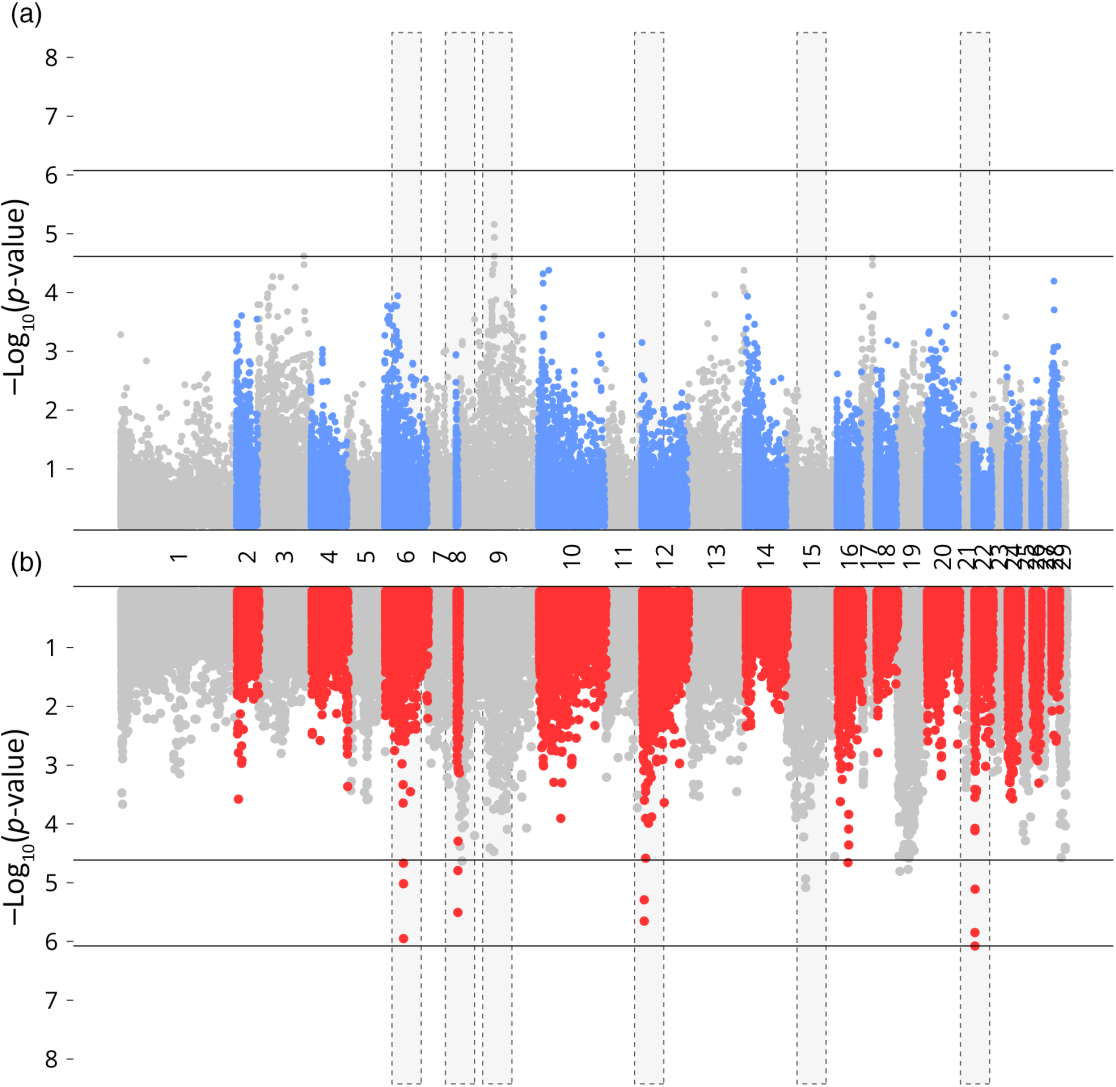
GWAS of WBW in loch (blue) and RAS (red) environments, regions with differential SNP association between environments highlighted in yellow. Lower and upper dashed line indicates chromosome and genome significance levels.

**FIGURE 3:**
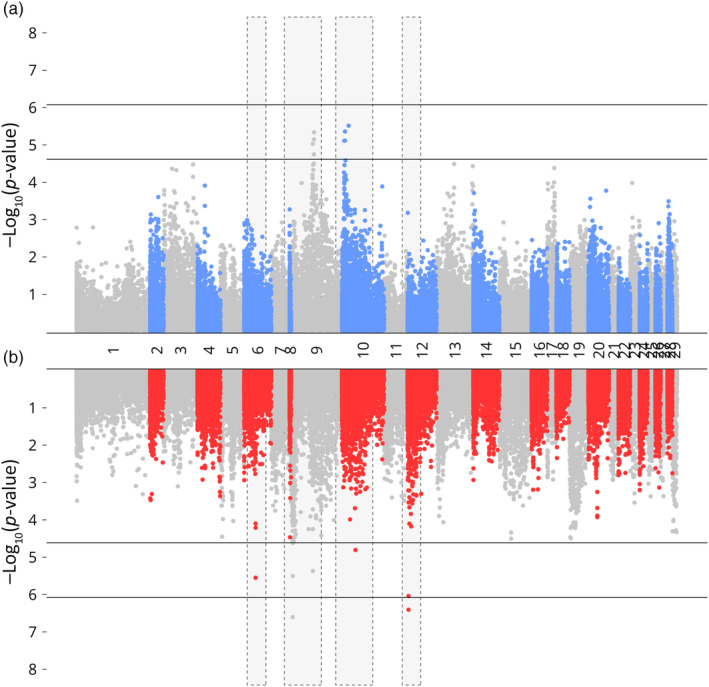
GWAS of length in loch (blue) and RAS (red) environments, regions with differential SNP association between environments highlighted in yellow. Lower and upper dashed line indicates chromosome and genome significance levels.

**FIGURE 4:**
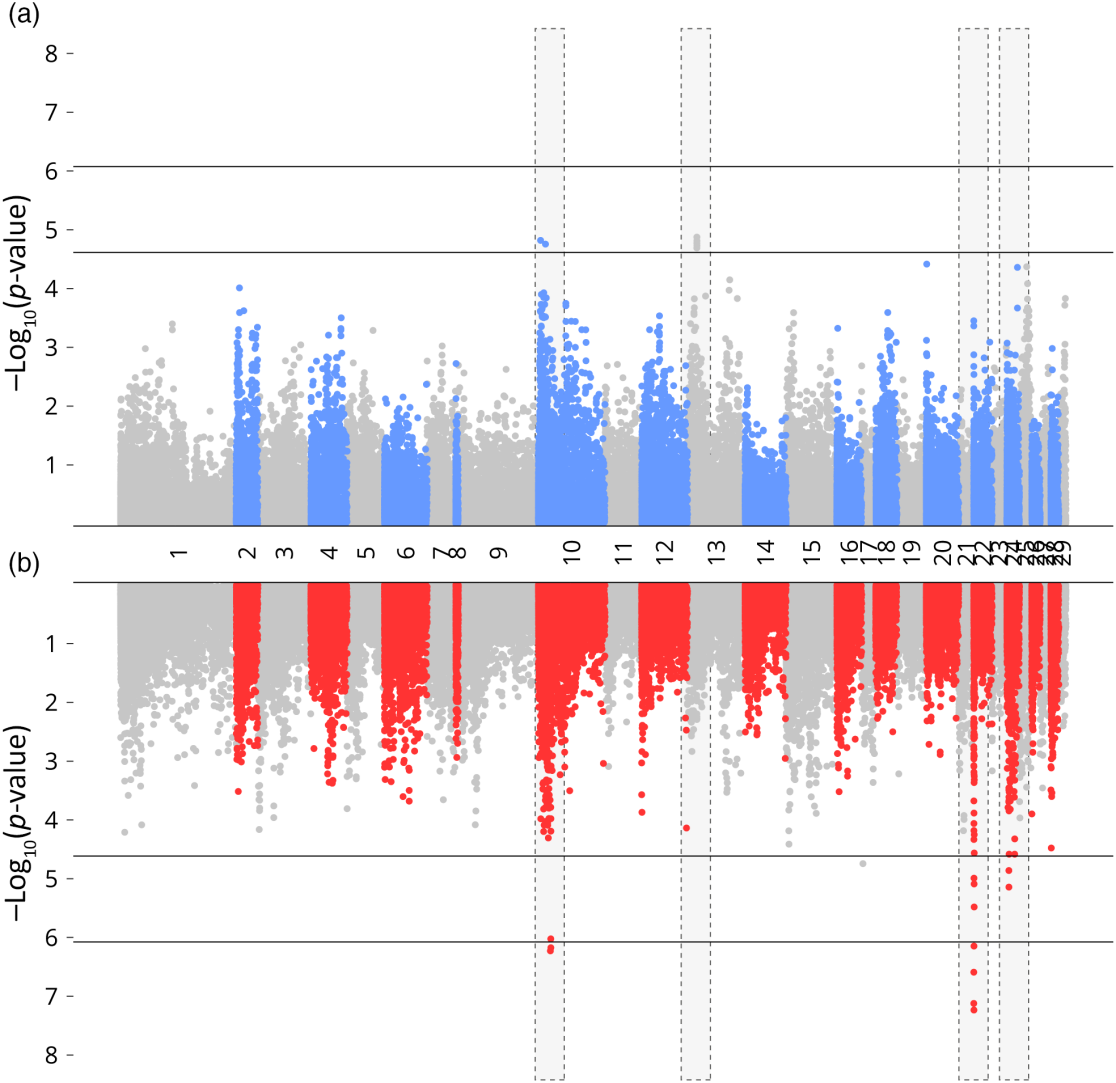
GWAS of condition factor in loch (blue) and RAS (red) environments, regions with differential SNP association between environments highlighted in yellow. Lower and upper dashed line indicates chromosome and genome significance levels.

The RAS environment exhibited a higher number of SNPs that had significant associations with growth traits compared to the loch environment, where no SNPs surpassed the genomic threshold (Table 3). For WBW, significant SNPs in the RAS population were identified on chromosomes 6, 8, 12, and 21, whereas they were found on chromosome 9 alone in the loch population (Figure 2). For length in RAS, these were observed on chromosomes 6, 8, 9, 10, and 12, with SNPs in the loch population also present on chromosomes 9 and 10 but with a higher level of significance (Figure 3). In the case of condition factor, while SNPs in the loch population exhibited an association with chromosome 13, regions on chromosomes 10, 24, and notably, chromosome 22 were identified in the RAS population (Figure 4). The Tables S6 and S7 provide details on the precise genomic locations.

**TABLE 3:** Number of SNPs found in significant association with growth rates at the genome and chromosome level; where WBW is whole body weight and K condition factor.

	Genome		Chromosome	
	RAS	Loch	RAS	Loch
WBW	0	0	15	2
Length	2	0	8	8
K	6	0	13	7

## 4 DISCUSSION

### 4.1 Phenotype and trait variances

According to our results, fish reared in the freshwater loch environment were both heavier and longer than their RAS counterparts, with sex having a secondary effect on growth measurements. However, RAS fish showed on average a greater condition factor. The available environmental information does not explain the differences seen in growth performance. The fish reared in the RAS population would be expected to have a higher growth rate as they were exposed to continuous light, greater oxygen saturation and higher water temperatures (Figure 1). Estimates of trait variance seen here fall in line with previously reported values for the coefficient of variation in salmon (Fishback et al., 2002; Gjedrem, 2000; Gjerde et al., 1994; Gjerde & Gjedrem, 1984; Gonzalez et al., 2022; Neira et al., 2004; Quinton et al., 2005). Reductions in trait variance can be achieved by husbandry manipulation, and the tighter environmental management offered by RAS is expected to increase consistency in family performance (Vu et al., 2021). Accordingly, compared to the loch environment studied here, RAS was less variable in terms of temperature and light exposure. Other variables did, however, show greater variability (pH and oxygen saturation) (Figure 1). Comparing these findings to other species, measurements of trait variance have also been lower when reared in recirculating systems compared to other husbandry environments (Sae‐Lim et al., 2014; Van Sang et al., 2020), yet other studies have been found to contradict this (Dupont‐Nivet et al., 2008, 2010; Li et al., 2019; Mas‐Muñoz et al., 2013).

While increasing uniformity via environmental management is particularly relevant in instances where phenotypic variance is largely driven by non‐genetic factors, when it is instead genetically underpinned, trait uniformity can alternatively be increased through selective breeding programs (Vu et al., 2021). This relates to ideas of robustness and micro‐environmental sensitivity, defined as an individual's ability to buffer against the effects of unknown biotic and abiotic disturbances within a single macro‐environment, as well as developmental or endogenous disturbances (de Souza Iung et al., 2020; Sonesson et al., 2013). Importantly, both are increasingly becoming targets for selection (Berghof et al., 2018; Sae‐Lim et al., 2015). Specifically, studying micro‐environmental sensitivity assumes a trait's residual variance as a proxy for uniformity before identifying its variance components. This allows estimates of breeding values and heritability for a trait's uniformity itself (Agha et al., 2018; Hill & Mulder, 2010). Yet, studies into micro‐environmental sensitivity are limited, as are practical examples of its selection (de Souza Iung et al., 2020; Garreau et al., 2008). Despite this, significant estimates of genetic variance and heritability for trait uniformity have been identified in salmonids (e.g. Janhunen et al., 2012; Sae‐Lim et al., 2017; Sonesson et al., 2013). Furthermore, in a comparison between body weight of *Litopenaeus vannamei* reared in RAS or low‐density earthen ponds, greater heritability estimates were found for body weight uniformity in the RAS environment (Garcia et al., 2021). This would suggest a heightened possibility of increasing uniformity through selection in RASs compared to other rearing environments. Further studies into micro‐environmental sensitivity could reveal if the differences seen here in trait variance between freshwater environments are underpinned by differences in genetic architecture and regulation of trait uniformity in addition to possible environmental effects.

### 4.2 Within environment genetic parameters

Within environments, heritability estimates for WBW and length ranged from moderate to low, all falling within the range reported previously for Atlantic salmon (Gjerde et al., 1994; Gonzalez et al., 2022; Khaw et al., 2021; Quinton et al., 2005; Rye & Refstie, 1995; Tsai et al., 2015; Yáñez et al., 2014). While the estimated heritability for the condition factor in the loch population fell within the range reported previously (Neira et al., 2004; Rye & Refstie, 1995), estimates of RAS were above this range (Table 1). In line with this, our results suggest the freshwater rearing environment effected heritability by increasing phenotypic and residual variation, leading to estimates in RAS of over twice that of the loch environment across traits (Table 1). Interestingly, (Dupont‐Nivet et al., 2010) also estimated lower trait heritability in freshwater body weight for European sea bass, *Dicentrarchus labrax*, reared in open sea cages or raceways, when compared closed RAS facilities.

This environmental difference in heritability appears a result of, firstly, a greater residual variance component in the loch environment (Table 1). As with the discussion of trait uniformity above, the smaller residual variance component in RAS is a hypothesised result of the husbandry control this environment permits. Conversely, the ambient environmental variability in the loch environment is suggested to have increased residual variance. Secondly (with the expectation of WBW), the RAS environment reported greater additive genetic variance component (Table 1) which will have also increased heritability estimates. Furthermore, as it accounts for differences in trait mean, *CGV* is often used to compare the genetic variance between environments. Values reported here reveal that the difference in *V*_*g*_ between environments is similarly seen in *CGV* where values reported were greater in RAS compared to loch environment, most clearly demonstrated in WBW. Interestingly, heterogeneous genetic variance and heritability between environments are additional indications of genotype–environments interactions to re‐ranking (Sae‐Lim et al., 2014).

In contrast, correlations within environments were largely similar between the RAS and loch populations (Table 2). Strongly positive residual and genetic correlations were seen between WBW and length, as expected, and previously recorded by other studies (Gjerde & Gjedrem, 1984; Rye & Refstie, 1995; Tsai et al., 2015). Positive genetic and residual correlations were also seen between WBW and condition factor, although they were slightly greater in the RAS compared to loch environment. An interesting point of difference was seen in the genetic correlation between condition factor and length. In line with previous studies, a weak negative genetic correlation was estimated between condition factor and length in the loch environment (Fishback et al., 2002; Neira et al., 2004; Rye & Refstie, 1995). Whereas in RAS, this correlation was weakly positive (Table 2), indicating selection on any one of the growth traits could act to increase the others as well, a desirable outcome.

### 4.3 Genotype–environment interactions

Here, we quantified GxE as the genetic correlation between the same trait measured in different environments. A range of thresholds have been suggested for genetic correlations as the boundaries for the severity of family re‐ranking (Navarro et al., 2009; Ponzoni et al., 2005). Estimates of GxE of growth traits measured here ranged from moderate to weak. Specifically, while the genetic correlation for both condition factor and length were above 0.7, for WBW it was 0.62 suggesting a significant level of re‐ranking. Results presented here should, however, take into consideration the variability of family size between environments.

The strength of GxE is often attributed to the degree of difference between environments, whereby the greater the environmental difference, the stronger the GxE identified (Mengistu et al., 2020; Nguyen et al., 2017; Sae‐Lim et al., 2015). Looking to compare the environment of freshwater lochs and RAS, evidence on the RAS specific environment has previously been reviewed, highlighting stocking density, temperature, light quality and quantity, water quality, as well as environmental stability (Ebeling & Timmons, 2012; Good & Davidson, 2016; Schumann & Brinker, 2020). Here differences were seen in temperature, oxygen saturation, lighting conditions and stocking density (Figure 1). Considering this, significant re‐ranking was previously reported in response to temperature (Hebert et al., 1998; Strait et al., 2020) and photoperiod (Stefansson et al., 1990) in salmonids, as well as oxygen saturation in tilapia (Mengistu et al., 2020), though others have instead seen high genetic correlations (Fishback et al., 2002; Hanke et al., 1989). Furthermore, in a cross‐continental study in rainbow trout, degree days and photoperiod were suggested as the main environmental variables driving significant re‐ranking between a nucleus population and varied production environments (Sae‐Lim et al., 2013, 2014). It is unclear from the environmental information available here what the causal factors could be driving the significant re‐ranking in WBW. It would be of benefit to perform GxE analysis with more environmental parameters recorded across environments, enabling similar analysis to Sae‐Lim et al. (2014). If probable causative environmental factors can be identified, targeted husbandry practices could be implemented to reduce differences between RAS and loch environments and in turn the magnitude GxE in WBW. Equally, it would be of interest to investigate the stability of GxE interactions within the RAS environment itself. Previous studies have shown genetic correlations to temporally fluctuate (e.g. Fernandes et al., 2019), and information on this may help to give context to the impact of GxE observed here between FW environments after only a short period of separate rearing.

In line with previous GWAS in Atlantic salmon, few SNPs were found in significant association with growth traits in either in environment, which combined with moderate heritability estimates indicates a pattern of polygenic regulation (e.g. Gutierrez et al., 2015; Tsai et al., 2015; Yoshida et al., 2017). However, in the RAS environment, a greater number of SNPs were found in significant association with traits of interest (Table 3). This aligns with the findings that residual compared to genetic variance explained a greater proportion of phenotypic variance in the loch population (Table 1). Our results also suggest environment specific differences in the trait associations. Of particular note is the region identified on chromosome 22 found in significant association with condition factor in the RAS population only (Figure 4).

### 4.4 Implications for aquaculture

Our results suggest that although producing smaller fish, the RAS environment significantly reduced phenotypic variance. This also translated into a higher heritability estimate in RAS, which suggests that there could be greater opportunity to apply selection in RAS.

While environmental parameters measured here did not reveal large differences between environments, significant re‐ranking was identified for WBW. The genetic correlation of this trait between environments fell below the 0.7 threshold (Sae‐Lim et al., 2013). Therefore, as production of Atlantic salmon smolts in RAS continues to increase, it may be advantageous to investigate introducing RAS specific growth into breeding goals, including further research into RAS specific markers identified in GWAS.

## 5 CONCLUSIONS

Salmon reared in a RAS production system, which, although smaller in terms of weight and length, had a higher condition factor and showed substantially less variation. A significantly smaller component of residual variance was also found in the RAS reared fish compared to the freshwater loch environment, translating into higher heritability estimates across all three traits. Importantly, significant re‐ranking was identified for body weight between environments, as were differences in the genetic associations with growth traits.

## FUNDING INFORMATION

M.J.T. was supported by the UK Biotechnology and Biological Science Research Council (BBSRC) EASTBIO Doctoral Training Partnership, and the University of Stirling. A.D.‐W. was partly funded by the BBSRC Institute Strategic Programme Grants BBS/E/D/30002276 and BB/X010945/1. The study was funded as part of the BBSRC/NERC project ROBUST‐SMOLT ‘Impact of early life history in freshwater Recirculation Aquaculture Systems on A. salmon robustness and susceptibility to disease at sea’ (BB/S004432/1). The funding bodies played no role in the design of the study and collection, analysis, and interpretation of data and in writing the manuscript.

## CONFLICT OF INTEREST STATEMENT

A.N. and H.M. are employed by Mowi Genetics AS and Mowi Scotland, respectively. R.H. is employed by Benchmark Genetics. The remaining authors declare that the research was conducted in the absence of any commercial or financial relationships that could be construed as a potential conflict of interest.

## INSTITUTIONAL REVIEW

Animal handling and collection in this study was carried out in accordance with the UK Animals (Scientific Procedures) Act 1986 Amended Regulations (SI 2012/3039) and the work was approved by the University of Stirling Ethics Committee (Animal Welfare and Ethics Review Board; AWERB 2021 4871 3775).

## Supporting information


Data S1

Data S2

Appendix S1

## Data Availability

All relevant data are within the paper and its supporting information files.
