# Supplementary material for: Assessing genotype–environment interactions in Atlantic salmon reared in freshwater loch and recirculating systems
Source: Evol Appl. 2024 Aug 9;17(8):e13751. doi: 10.1111/eva.13751 (PMC11310769; doi:10.1111/eva.13751)
Supplement: Supplementary file 3 — Appendix S1 [file EVA-17-e13751-s002.pdf]

Assessing genotype-environment interactions in Atlantic salmon reared in freshwater loch and recirculating systems

Mette J. Tollervey[1], Michaël Bekaert[1,2,*], Agustín Barría González[3], Saif Agha[3,4], Ross D. Houston[5], Andrea Doeschl-Wilson[3], Ashie Norris[6], Herve Migaud[1,7], and Alejandro P. Gutierrez[1]

[1]**Institute of Aquaculture, University of Stirling, Stirling FK9 4LA, UK**
[2]**Cooke Aquaculture Scotland, Avondale House, Strathclyde Business Park, Bellshill, ML4 3NJ, UK**
[3]**Roslin Institute, The University of Edinburgh, Easter Bush Campus, EH25 9RG, UK**
[4]**Animal Production Department, Faculty of Agriculture, Ain Shams University, Shubra Alkhaima, Cairo, 11241, Egypt**
[5]**Benchmark Genetics, Edinburgh Technopole, Edinburgh, EH26 0GB, United Kingdom.**
[6]**Mowi Genetics AS, Bergen, 5035, Norway**
[7]**Mowi Scotland, Glen Nevis Business Park, Fort William, PH33 6RX, UK**

Corresponding author:
Michaël Bekaert[1]

Email address: michael.bekaert@stir.ac.uk

SUPPLEMENTARY MATERIAL

Supplementary Data S1 Anonymised genotypes of the 1,942 samples. (VCF)

Supplementary Data S2 Metadata of the 1,942 samples, parentage, length, weight, sex, environment. etc. (CSV)

Supplementary Table S1. Summary of environment variable changes. Mean, standard deviation and range of additional water quality parameters recorded in the RAS environment during the period of separate rearing. * below the measurable range.

Parameter	Mean (SD)	Range
O_2 saturation (%)	101.09 (2.65)	94.00 - 107.00
pH	6.96 (0.16)	6.53 - 7.48
Temperature (°C)	13.21 (0.94)	11.30 - 14.70
Turbidity (NTU)	1.07 (0.85)	0.08 - 3.88
TAN (mg/L)	0.0707 (0.0720)	0* - 0.28
Nitrite (mg/L)	0.0337 (0.0529)	0* - 0.2300
Nitrate (mg/L)	235.61 (64.18)	90.00 - 357.00
Toxic ammonia (NH_3) (mg/L)	0.00022 (0.00027)	0* - 0.00123
CO_2 (mg/L)	5.57 (1.81)	3.75 - 12.50
Total alkalinity (mg/L)	108.30 (45.77)	30.00 - 210.00
Calcium hardness (mg $CaCO_3$/L)	30.30 (18.22)	0* - 58.00
Total hardness (mg $CaCO_3$/L)	61.80 (26.21)	5.00 - 115.00

Supplementary Table S2. Number of SNPs removed in quality control for parental and offspring genotypes.

	Step	Parents	Offspring
	Start	55,357	65,774
SNP removed	Dup/chr30	506	785
	<10% SNP	106	1146
	<10% individual	0	0
	HWE	54	3,143
	MAS	1,202	1,122
	End	53,489	59,578

Supplementary Table S3. genomic distribution of common SNPs retained for analysis, including minimum and maximum SNP position, SNP number, and SNP coverage, per chromosome.

Chr.	SNP number	Coverage (kb)
1	3,401	158,976
2	1,210	72,910
3	2,139	92,338
4	1,846	82,398
5	1,847	80,317
6	1,842	87,014
7	1,317	57,626
8	396	26,233
9	2,532	141,543
10	2,508	116,086
11	1,773	93,888
12	1,815	91,863
13	2,281	107,731
14	2,038	93,886
15	1,949	103,896
16	1,554	87,455
17	1,091	57,656
18	1,193	70,695
19	1,467	82,833
20	1,846	86,732
21	1,153	57,906
22	1,355	63,060
23	1,303	49,692
24	1,143	48,558
25	1,037	51,339
26	900	47,201
27	1,122	43,591
28	922	39,329
29	781	42,455

Supplementary Table S4. Parameter estimates from genomic analysis of trait (whole body weight (WBW, g), length (cm), and condition factor (K)) architecture performed using pedigree (*A* matrix) information only.

	WBW (g)		Length (cm)		K	
	RAS	Loch	RAS	Loch	RAS	Loch
V_r (SE)	173.70 (18.96)	569.89 (42.10)	0.85 (0.09)	2.35 (0.16)	$0.35 \cdot 10^{-2}$ ($0.38 \cdot 10^{-3}$)	$1.00 \cdot 10^{-2}$ ($0.67 \cdot 10^{-3}$)
V_g (SE)	129.35 (26.64)	164.11 (45.35)	0.52 (0.11)	0.44 (0.15)	$0.28 \cdot 10^{-2}$ ($0.54 \cdot 10^{-3}$)	$0.16 \cdot 10^{-2}$ ($0.61 \cdot 10^{-3}$)
h^2 (SE)	0.42 (0.08)	0.22 (0.06)	0.38 (0.07)	0.16 (0.05)	0.44 (0.07)	0.13 (0.05)

Supplementary Table S5. Parameter estimates from genomic analysis of trait (whole body weight (WBW, g), length (cm), and condition factor (K)) architecture performed using genomic relationship matrix (*GRM*) information only.

	WBW (g)		Length (cm)		K	
	**RAS**	**Loch**	**RAS**	**Loch**	**RAS**	**Loch**
V_r (SE)	171.41 (13.65)	588.48 (35.65)	0.81 (0.06)	2.32 (0.14)	$0.37{\cdot}10^{-2}$ ($0.27{\cdot}10^{-3}$)	$1.00{\cdot}10^{-2}$ ($0.60{\cdot}10^{-3}$)
V_g (SE)	131.34 (21.86)	133.63 (34.43)	0.57 (0.10)	0.44 (0.13)	$0.23{\cdot}10^{-2}$ ($0.40{\cdot}10^{-3}$)	$0.15{\cdot}10^{-2}$ ($0.52{\cdot}10^{-3}$)
h^2 (SE)	0.43 (0.06)	0.18 (0.05)	0.41 (0.06)	0.16 (0.04)	0.38 (0.05)	0.14 (0.04)

Supplementary Table S6. Genomic position (chromosome and base-pair location), allelic polymorphism (A and B), minor allelic frequency, effect (b) and its standard error (SE) and significance ($-log_{10}(P)$). Coordinates based on *S. salar* Ssal_3.1 genome assembly.

Chr.	bp	A	B	MAF	b	SE	$-log_{10}(P)$	Trait	Group
9	21,914,899	G	T	0.45	0.28	0.055	6.60	length	All
10	45,990,818	T	C	0.50	-0.02	0.004	6.23	K	All
10	47,402,831	A	C	0.50	-0.02	0.004	6.17	K	All
12	19,883,879	G	C	0.42	0.32	0.062	6.41	length	All
22	334,834	C	T	0.12	0.03	0.006	7.12	K	All
22	2,496,554	G	T	0.11	0.03	0.006	6.59	K	All
22	5,723,842	T	G	0.16	0.03	0.005	7.23	K	All
22	6,138,261	G	T	0.19	0.02	0.005	6.15	K	All

Supplementary Table S7. From GWAS, SNPs found in significant association with whole body weight (WBW), length (cm) and condition factor (K) at the chromosome specific 5% significance level. This is for the pooled population (overall) as well as RAS and loch specific populations. Coordinates based on *S. salar* Ssal_3.1 genome assembly.

Chr.	bp	A	B	MAF	b	SE	$-log_{10}(P)$	Trait	Group
6	55,995,298	A	C	0.46	-3.60	0.85	4.63	weight	ras
6	56,004,795	A	G	0.45	-3.87	0.88	4.98	weight	ras
6	56,008,205	A	G	0.37	-0.30	0.06	5.55	length	ras
6	56,008,205	A	G	0.37	-4.57	0.94	5.91	weight	ras
8	13,005,872	C	A	0.47	-4.02	0.87	5.47	weight	ras
8	13,694,966	T	C	0.25	4.38	1.02	4.76	weight	ras
9	21,914,899	G	T	0.45	0.28	0.05	6.60	length	ras
9	22,226,040	G	C	0.42	-0.26	0.06	5.50	length	ras
9	22,481,480	C	T	0.39	-0.23	0.06	4.62	length	ras
9	94,673,165	C	T	0.36	-0.39	0.09	5.01	length	loch
9	94,766,724	A	G	0.28	-0.31	0.07	5.37	length	ras
9	96,767,787	C	T	0.28	-7.00	1.56	5.14	weight	loch
9	96,782,701	A	G	0.18	-0.47	0.11	4.73	length	loch
9	96,797,435	T	G	0.18	-0.48	0.11	5.13	length	loch
9	96,811,937	A	G	0.18	-7.87	1.80	4.92	weight	loch
9	96,811,937	A	G	0.18	-0.50	0.11	5.32	length	loch
10	16,843,445	C	A	0.17	-0.03	0.01	4.82	K	loch
10	21,376,501	G	A	0.09	0.64	0.14	5.10	length	loch
10	24,437,130	C	G	0.11	0.60	0.13	5.34	length	loch
10	24,454,770	G	A	0.24	0.42	0.09	5.1 0	length	loch
10	30,476,258	G	T	0.22	-0.03	0.01	4.76	K	loch
10	40,854,514	T	C	0.11	0.61	0.13	5.50	length	loch
10	45,990,818	T	C	0.5	-0.02	0.00	6.23	K	ras
10	47,398,720	G	A	0.5	-0.02	0.00	6.02	K	ras
10	47,402,831	A	C	0.5	-0.02	0.00	6.17	K	ras
10	68,374,806	G	A	0.3	0.27	0.06	4.81	length	ras
12	19,883,879	G	C	0.42	4.32	0.92	5.62	weight	ras
12	19,883,879	G	C	0.42	0.32	0.06	6.41	length	ras
12	19,899,766	A	G	0.43	4.10	0.90	5.25	weight	ras
12	19,899,766	A	G	0.43	0.30	0.06	6.04	length	ras
13	40,473,884	G	T	0.13	0.03	0.01	4.69	K	loch
13	40,474,212	C	T	0.13	0.04	0.01	4.77	K	loch
13	40,475,549	T	G	0.13	0.04	0.01	4.88	K	loch
13	40,483,278	G	A	0.14	0.04	0.01	4.81	K	loch
13	40,484,059	C	A	0.13	0.04	0.01	4.73	K	loch
15	62,331,139	A	G	0.38	-3.64	0.83	4.90	weight	ras
15	62,332,972	A	G	0.38	-3.70	0.83	5.04	weight	ras
16	43,224,956	A	G	0.32	-3.85	0.91	4.62	weight	ras
17	13,200,053	T	C	0.07	-0.03	0.01	4.74	K	ras
19	18,280,978	T	C	0.51	-3.60	0.84	4.77	weight	ras
19	49,828,526	A	G	0.16	5.21	1.22	4.74	weight	ras
22	334,834	C	T	0.12	6.56	1.34	6.04	weight	ras
22	334,834	C	T	0.12	0.03	0.01	7.12	K	ras
22	2,496,554	G	T	0.11	6.42	1.34	5.81	weight	ras
22	2,496,554	G	T	0.11	0.03	0.01	6.59	K	ras
22	5,723,842	T	G	0.16	5.15	1.16	5.07	weight	ras
22	5,723,842	T	G	0.16	0.03	0.01	7.23	K	ras
22	6,138,261	G	T	0.19	0.02	0.00	6.15	K	ras
22	6,163,393	A	G	0.35	0.02	0.00	4.99	K	ras
22	7,146,753	G	A	0.28	0.02	0.00	5.09	K	ras
22	7,824,331	C	T	0.15	0.03	0.01	5.48	K	ras
24	15,406,969	C	T	0.38	-0.02	0.00	4.86	K	ras
24	15,418,645	C	T	0.38	-0.02	0.00	5.14	K	ras

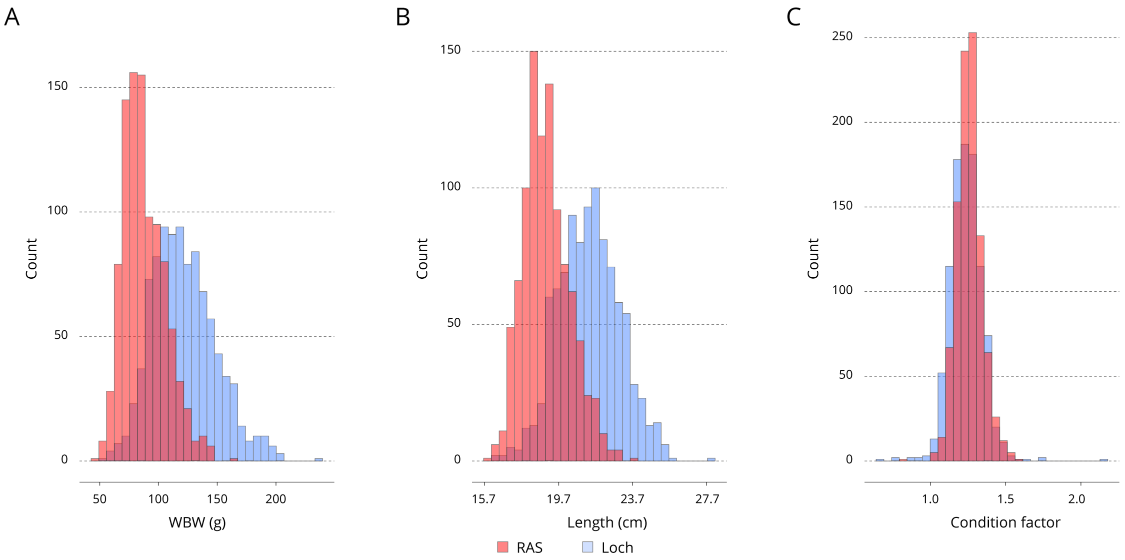

Supplementary Figure S1. histogram of whole body weight (WBW, g), length (cm), and condition factor (K), split between RAS (red) and loch (blue) populations.
